# ToRaRI (Tofacitinib in Rheumatoid Arthritis a Real-Life experience in Italy): Effectiveness, safety profile of tofacitinib and concordance between patient-reported outcomes and physician's global assessment of disease activity in a retrospective study in Central-Italy

**DOI:** 10.1007/s10067-023-06836-w

**Published:** 2023-12-23

**Authors:** Francesco D’Alessandro, Massimiliano Cazzato, Elenia Laurino, Riccardo Morganti, Marco Bardelli, Bruno Frediani, Claudia Buongarzone, Gianluca Moroncini, Serena Guiducci, Laura Cometi, Maurizio Benucci, Francesca Ligobbi, Daniela Marotto, Marta Mosca

**Affiliations:** 1 Rheumatology Unit, University of Pisa, Pisa, Italy; 2 Section of Statistics, University of Pisa, Pisa, Italy; 3 Rheumatology Unit-Department of Medicine, Surgery and Neurosciences-University Hospital Siena, Siena, Italy; 4 Internal Medicine Residency Programme, Marche Polytechnic University, Ancona, Italy; 5 Clinica Medica, Marche Polytechnic University Hospital, Ancona, Italy; 6 Department of Experimental and Clinical Medicine, Division of Rheumatology, University of Florence, Florence, Italy; 7 Rheumatology Unit, S.Giovanni Di Dio Firenze Hospital, Florence, Italy; 8 Reumatology Unit Asl Gallura Olbia, Olbia, Italy

**Keywords:** Rheumatoid arthritis, Jak inhibitors, Patient-reported outcomes, Physician's global assessment

## Abstract

**Introduction:**

The use of Janus Kinase Inhibitors (JAK-Is) in rheumatoid arthritis (RA) has entered in daily practice. In consideration of ORAL-Surveillance trial and the new EULAR recommendations, real-world data are needed to assess Jak-Is safety and effectiveness. The multicenter study presented here aimed to evaluate effectiveness and safety of tofacitinib in a real-life cohort.

**Methods:**

A retrospective analysis was performed from September 2021 to December 2022. Data were collected when tofacitinib was started (T0) and after 3 (T3), 6 (T6) and 12 (T12) months of treatment. The primary objective was to analyze the efficacy and safety of tofacitinib. Safety was assessed by recording adverse events (AEs) with and without discontinuation.

The secondary objective was to assess the difference between Patient-Reported Outcomes (PROs) and Physician's Global Assessment of disease activity (PhGA).

**Results:**

122 patients were included in the study from the following rheumatology Centers: Pisa, Ancona, Florence (two Centers), Siena, and Sardinia. A statistically significant improvement in DAS-28-CRP, CDAI and SDAI score was observed at T3, T6, compared to baseline (*p* < 0.001). Improvement was confirmed in patients who reach T12. Patients naïve to bDMARDs showed a shorter remission time and higher remission rates. There was also a statistically significant improvement in PROs compared to baseline (*p* < 0.001). The improvement was rapid and was consistent with PhGA. The 12-month retention rate for tofacitinib was 89.35%. Reasons to stop tofacitinib were: insufficient response (7), gastrointestinal symptoms (2), infection (1), malignancy (1), Zoster (1), pruritus sine materia (1).

**Conclusions:**

Tofacitinib is safe and effective in our RA cohort. It induces higher remission rates in patients naive to bDMARDs, suggesting that there may be a benefit using it as first-line therapy. Additionally, improvement in PROs was consistent with PhGA scores, demonstrating how tofacitinib affects both the objective and subjective components of disease activity.

**Table:** 

**Key Points** 1. *JAK inhibitors are considered at a similar level as biologic agents in terms of effectiveness.* 2. *After ORAL-Surveillance results, real-world data are needed to assess the benefit/risk profile of Jaki.* 3. *Disagreement between patients and physicians has been previously reported with biologic therapy among patients with rheumatoid arthritis, with patients rating disease activity higher than physicians.* 4. *Jak inhibitors could reduce this discrepancy, due to their mechanism of action.*

## Introduction

Rheumatoid arthritis (RA) is a chronic autoimmune disease characterized by local and systemic inflammation driven by the interaction between immune cells and soluble mediators, which is clinically manifested by joint and extra-articular involvement [1]. The treatment target is to characterize clinical phenotype by acting on the cytokine pathways to stop the inflammatory process, the progression of joint damage and to improve the patient's quality of life. In clinical practice, we use composite indices of disease activity, such as DAS-28, CDAI and SDAI in order to have an objective evaluation of the patient and to monitor the specific clinical progress [2].

The Janus kinase (JAK) signal transducers and activators of transcription (STAT) proteins constitute the JAK-STAT pathways, which represent an important pathogenic mechanism in RA [3].

JAK inhibitors (JAKis) are small-molecule drugs that interfere with the activation of JAKs, a family of enzymes implicated in the signaling of leukocytes. JAK signaling plays an essential role in immune cell generation, differentiation and responses [4]. By inhibiting these signaling mechanisms, JAK inhibitors affect immune activation that is essential for RA [5].

Therefore, JAKis have emerged as an important new class of oral therapy in RA. Baricitinib (4 or 2 mg daily), Tofacitinib (5 mg twice daily), Upadacitinib (15 mg daily) and Filgotinib (200 or 100 mg daily) are currently approved for the treatment of RA by the US Food and Drug Administration (all except Filgotinib) and the European Medicines Agency [6].

In 2019, JAK inhibitors were recommended as a second line treatment for RA, at a similar level as bDMARDs (biological Disease-modifying Anti-rheumatic drugs) in terms of effectiveness and safety. However, in 2023, EULAR published new recommendations for the management of RA. Based on the warning of cardiovascular and malignancy risks[7] highlighted by ORAL-Surveillance trial results, the assessment of cardiovascular risk factors (age over 65 years, history of current or past smoking, other cardiovascular risk factors), thromboembolic events and malignancies are important to assess when intending to prescribe a JAK-is [8]. Nevertheless, long-term extension trials and registries did not confirm the results of the ORAL-Surveillance trial [9, 10].

Different analyses established the effectiveness of JAK-is, with a comparable safety profile among them [11]*.*

JAK-is have shown efficacy in placebo-controlled trials, either as monotherapy or in combination with csDMARDs [12–14], but in consideration of the recent safety issues there is a need for real-world data.

The primary objective of this multicenter Italian study was to assess the effectiveness, as measured by DAS28, CDAI and SDAI, and safety of Tofacitinib during a 12-months follow up in patients with RA treated in a real-world setting.

The secondary objectives were to assess the difference between Patient-Reported Outcomes (PROs) and Physician's Global Assessment of disease activity (PhGA), gender variances and the efficacy in relation to the serological profile.

## Materials and methods

A retrospective multicenter study was performed; data were obtained in 6 Italian tertiary rheumatology centers: Pisa, Ancona, Florence (two Centers), Siena and Sardinia. A single comprehensive retrospective database was created for all RA patient meeting the 2010 ACR-EULAR criteria [15] treated with Tofacitinib.

### Study design

The study was performed in accordance with the ethical principles of the Helsinki Declaration and according to the principles of good clinical practice.

The retrospective analysis was conducted from September 2021 to December 2022. Data were collected when tofacitinib was started (T0) and after 3 (T3), 6 (T6) and 12 (T12) months of treatment.

We included age, sex, disease duration, smoking habits, antibody profile with rheumatoid factor (RF) and anti-cyclic citrullinated peptide antibody (ACPA), episodes of Zoster, malignancies, cardiovascular profile, Major Adverse Cardiac Events (MACE: nonfatal stroke, nonfatal myocardial infarction, and cardiovascular death) [16] and thromboembolic events. Moreover, we collected history and current cDMARDs (conventional Disease-modifying Anti-rheumatic Drugs), bDMARDs, tsDMARDs (targeted synthetic Disease-modifying Anti-rheumatic Drugs) and steroid treatment as prednisone equivalent daily glucocorticoid dose.

We assessed the disease activity including tender and swollen joint count on 68 and 66 joints, C-reactive protein (CRP), erythrocyte sedimentation rate (ESR), disease activity score calculated with C-reactive protein (DAS28-CRP), Clinical Disease Activity Index (CDAI) and simplified disease activity index (SDAI). PROs as patient's assessment of disease activity (PtGA) on a 0-100 mm scale, general health status (GH; 0–100 visual analog scale) and physician's global assessment of disease activity (PhGA) were recorded at each visit.

Adverse events (AEs) were recorded at T3, T6, and T12 and classified into blood count alterations, MACE, infectious, gastrointestinal, thromboembolic or neoplasia events. Moreover, the reasons for JAK-is discontinuation were classified as lack of efficacy, adverse event, or loss to follow-up.

### Statistical analysis

Descriptive statistics were used to describe the basic features of the population and continuous data are displayed using the mean and standard deviation (SD). For statistical analysis, variant analysis for repeated measurements and Pearson correlation analysis were used. The χ2 (chi-square) was used to check for statistical differences compared to categorical outcomes.

Discrepancy between PtGA e PhGA (ΔPGA) was calculated as PtGA minus PhGA and classified as discordant when ≥|20 mm| [17, 18].

Discrepancy between GH e PhGA (ΔGH) was calculated as GH minus PhGA and classified as discordant when ≥|20 mm|

Concordance was calculated as patient percentage with ΔPGA or ΔGH < 20 mm.

A secondary analysis was made to assess the correlation between the DAS-28 and PtGA, the variables clinically relevant in the researchers’ perspective, were included in multivariable linear regression analysis to identify determinants for DAS-28.

Correlations between DAS-28, with other variables was assessed through Pearson correlation coefficient (*r*) and comparison between groups through t-test. *r* values < 0.40 were considered poor, 0.40–0.59 moderate and ≥ 0.60 very good.

A *p*value < 0.05 was considered statistically significant. Statistical analysis was performed by means of the MedCalc statistical software.

## Results

### Baseline demographics and clinics

122 patients treated with tofacitinib 5 mg twice daily were included in our study.

The majority of patient were females (76%). At baseline, the average age at initiation of tofacitinib was 62.3 ± 12.8 (years ± SD), and the average duration of disease was 12.96 ± 10.7 (years ± SD). RF and ACPA were positive in 81 (68.1%) and 72 (59%) patients, respectively. The baseline mean disease activity was moderate to high measured by DAS-28 CRP (4,30 ± 0,88), CDAI (22,06 ± 8,1) and SDAI (24.64 ± 8,6) (Table 1).

**Table 1:** Characteristics of the population at baseline. Statistics: frequency (%) or Standard Deviation (sd)

Number	122
F/M	93/29
Age, years	62.3 (± 12.8)
Disease duration	12.97 (± 10.7)
Smoking, n (%)	25 (22.3%)
RF, positive	81 (68.1%)
Anti-CCP, positive	72 (59%)
Dyslipidemia, n (%)	47 (38.5%)
Statin, yes	14 (11.47%)
Cardiovascular disease, n (%)	17 (13.93%)
MACE, n (%)	3 (2.45%)
Previous malignancies, n (%)	7 (5,73%)
Previous Zoster episodes, n (%)	5 (4,09%)
bDMARDs failure, n (%)	86 (71.7%)
tsDMARD naïve, n (%)	119 (97.5%)
cDMARD, n (%)	44 (36%)
Steroids, yes	51 (41.8%)
ESR, mm/h	30.07 (± 18.8)
CRP, mg/dl	1.068 (± 1.75)
DAS-28 CRP	4.30 (± 0.88)
CDAI	22.06 (± 8.1)
SDAI	24.64 (± 8.6)
Tender joint count	5.87 (± 4.2)
Swollen joint count	3.80 (± 3.7)
Patient's assessment of disease activity (PtGA)	65.01 (± 17.6)
General health status (GH)	61.8 (± 18.89)
Physician's global assessment of disease activity (PhGA)	59.62 (± 16.8)

At baseline, the cardiovascular risk was assessed: 47 patients were dyslipidemic (38.5%), and 14 on statin therapy (11,47%), 17 patients had cardiovascular disease (13.93%) and three had MACE (2.45%). Moreover, 25 patients (22.3%) were current smokers, while 5 (4.5%) were past smokers.

Before beginning Tofacitinib therapy, 86 (71.7%) patients were taking bDMARDs, of these 65 (77%) had failed at least 2, while only 3 patients had been treated previously with a different JAK-is. At baseline, 39 patients (32%) were administered MTX (mean dosage 12.4 mg weekly), two were administered leflunomide, three were administered sulfasalazine and eight were administered hydroxychloroquine. Moreover, 51 patients (41.8%) reported a combined glucocorticoid therapy (mean dosage equivalent to 6.45 mg prednisone daily).

#### Effectiveness

Three months after beginning treatment with Tofacitinb, a statistically significant improvement was observed in all evaluated activity indices, clinimetric scores and PROs and was confirmed during the follow-up evaluations. The Table 2 shows the results after 3 and 6 months of treatment:

**Table 2:** Improvement and comparison of DAS-CRP, CDAI, SDAI, TJC, SJC, VAS, PhGA, GH at 3- and 6-months follow-up after JAK-is treatment starts

	Baseline (mean ± SD)	T3 (mean ± SD)	T6 (mean ± SD)	*p*-value
DAS 28-CRP	4.30 (± 0.88)	3.03 (± 0.88)	2.65 (± 0,93)	< 0,001
CDAI	22.06 (± 8.1)	11.30 (± 7.30)	9.32 (± 6.62)	< 0,001
SDAI	24.64 (± 8.6)	13.49 (± 7.80)	9.75 (± 6.84)	< 0,001
TJC	5.87 (± 4.2)	1.87 (± 2.1)	1,63 (± 2.7)	< 0,001
SJC	3.80 (± 3.7)	1.08 (± 1.33)	1.04 (± 1.78)	< 0,001
GH	65.01 (± 17.6)	40 (± 19.33)	35.30 (± 18.99)	< 0,001
PtGA	59.62 (± 16.8)	34.06 (± 17.86)	31.04 (± 16.18)	< 0,001
PhGA	61.8 (± 18.89)	39.39 (± 17.43)	33.65 (± 20.37)	< 0,001

A statistically significant improvement in DAS-28-CRP score was observed at T3, T6, compared to baseline (*p* < 0.001). At T3 and T6, 43 (23.5%) and 53 (43.4%) patients achieved remission, respectively, while 28 patients were in low disease activity (LDA) at both time points.

**Fig. 1:**
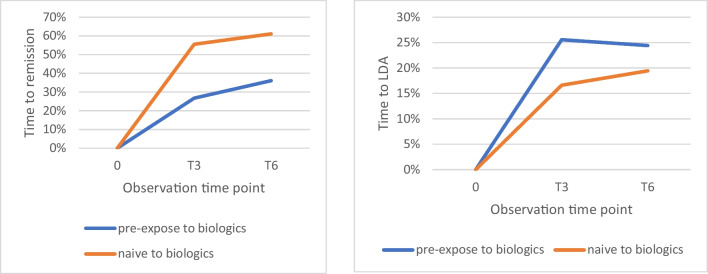
Disease activity: Time to DAS-28-CRP remission (left panel) and LDA (right panel) for all RA patients treated with tofacitinib. Patients previously exposed to biologic agent are shown in blue, while patients naïve to biologics in red

Time to DAS-28-CRP remission was shorter in patients naïve to biologics: at T3 55,5% naïve patients reached remission, compared with 26,74% of pre-exposed biologics (Fig. 1).

Furthermore, at T6 remission rates under tofacitinib were higher in patients naïve to biologics compared to patients who had been previously exposed (61.10% versus 36%).

Evaluation of CDAI at T3 and T6 revealed that 8 (6.5%) and 17(13.93%) patients achieved remission, whereas those in LDA were 50 (40.9%) and 51 (41.8%).

Evaluation of SDAI at T3 and T6 confirmed the above values, in fact 12 (9.8%) and 20 (16.3%) patients achieved remission respectively, whereas those in LDA at T3 and T6 were 37 (30.3%) and 52 (42.6%).

Moreover, there was a rapid and marked improvement in the tender and swollen joints count. Evaluation of TJC showed 1.87 (± 2.1) and 1,63 (± 2.7) tender joints at T3 and T6 respectively. The SJC showed the same evolution, in fact evaluation at T3 and T6 revealed 1.08 (± 1.33) and 1.04 (± 1.78) swollen joints.

Improvement was confirmed in patients who reach T12, as measured by DAS28-CRP (2.12 ± 1.07), CDAI (5.8 ± 5.9) and SDAI (5.9 ± 5.96) scores.

Regarding the use of glucocorticoids, 21 patients (out 51 at baseline) discontinued steroids at T3. At T6 only 23 patients continued glucocorticoids, achieving a mean dose of prednisone of 5.2 mg at T12.

A statistically significant correlation between higher number of bDMARDs failures and higher disease activity at T3, T6 and T12 was found.

### Patient-Reported outcomes versus physician's global assessment of disease activity

We found a statistically significant improvement in all PROs, with a rapid improvement in the first three months of therapy and a slight improvement in the following months.

At T3, the concordance between PtGA and PhGA was 72%, and the concordance between GH and PhGA was 79%.

At T6 the concordance between PtGA and PhGA increased to 78% and between GH and PhGA to 90% (See Table 3).

**Table 3:** Concordance between PROs and PhGA during the first six months

	Number of patients	Concordance %
PtGA and PhGA at T3	116	72%
GH and PhGA at T3	118	79%
PtGA and PhGA at T6	110	78%
GH and PhGA at T6	112	90%

Specifically, PtGA value reduced from 59.62 (± 16.8) at baseline, to 34.06 (± 17.86) at T3, up to 31.04 (± 16.18) at T6. While GH at the beginning resulted 65.01 (± 17.6) after three months was 40 (± 19.33) and at T6 was 35.30 (± 18.99).

PhGA showed the same improvement, and the results are consistent with all PROs: at baseline was 61.8 (± 18.89) at T3 was 39.39 (± 17.43) and at T6 was 33.65 (± 20.37).

#### Multivariable linear regression: Correlation between DAS-28 and PtGA at T3 and T6

In the multivariate analysis at T3 the dependency between DAS-28 and PtGA was moderate (*r* = 0.37, *p* < 0.001), also at T6 the correlation between the two data was confirmed (r = 0, 55; *p* < 0.001) (Table 4).

**Table 4:** Multivariable linear regression models for DAS-18 at T3 and T6

	B (95%CI)	95% CI—lower	95% CI—upper	parzial Pearson's r	*p*-value
Dependent variable: DAS28 T3					
PtGA-T3	0,021	0,011	0,031	0,437	< 0,001
Sex	0,177	-0,273	0,626	0,080	0,437
Age	-0,006	-0,022	0,009	-0,092	0,403
Diasease duration	-0,003	-0,024	0,019	-0,027	0,812
RF, positive	0,151	-0,243	0,545	0,078	0,448
Anti-CCP, positive	0,130	-0,256	0,516	0,071	0,505
Constant	2,333	1,423	3,244		< 0,001
Dependent variable: DAS28 T6					
PtGA-T6	0,024	0,015	0,033	0,555	< 0,001
Sex	-0,065	-0,528	0,398	-0,030	0,780
Age	-0,005	-0,021	0,010	-0,074	0,493
Diasease duration	0,010	-0,011	0,031	0,106	0,359
RF, positive	-0,135	-0,552	0,282	-0,066	0,521
Anti-CCP, positive	0,084	-0,310	0,478	0,045	0,673
Constant	2,281	1,336	3,226		< 0,001

DAS-28 showed no further correlation with the other clinimetric and demographic data.

#### Safety

During the 12-months follow-up period a total of 13 (10.65%) patients discontinued tofacitinib: 7 due to insufficient response and 6 to adverse events (AEs).

Specifically, at T3 two patients stopped for primary failure, one stopped for herpes zoster reactivation, one for gastrointestinal intolerance, one for severe respiratory infection, one for development of Large B-cell lymphoma. At T6 additional five patients have discontinued therapy: three for failure, one for gastrointestinal intolerance, one for pruritus sine materia; at T12 only two patients dropped out due to loss of drug efficacy (Table 2).

Thirty-two AEs without discontinuation were recorded. Blood count changes were the most common, occurring in eleven patients (9.01%), especially at T3 with anemia and lymphocytopenia. Herpes zoster reactivation was observed in eight patients (6.5%) in the first six months, no reactivation was registered at T12; while in twelve months, five serious infections were reported. Five other patients reported gastrointestinal intolerance, especially in the first three months of therapy (4.09%). During the study, two MACE (one stroke and one myocardial infarction) and one TIA (transient ischemic attack) were observed. No DVT or deaths were observed.

Notably, patients with cardiovascular disease and malignancies at baseline showed a greater number of adverse reactions at T3 (*p* < 0.005) and T6 (*p* = 0.057) respectively.

In addition, we did not find statistically significant correlations in adverse reactions when patients were categorized according to the following variables: sex, age, disease duration, serological profile, smoking habits, previous zoster episodes, dyslipidemia, current treatment with steroid, current cDMARDs use (Table 5).

**Table 5:** AEs description during twelve months observation

	AEs without discontinuation	AEs causing interruption
Malignancy	0	1
MACE	2	0
DVT	0	0
Death	0	0
Thrombocytosis	1	0
Neutropenia	2	0
Lymphopenia	3	0
Anemia	5	0
Zoster	8	1
Gastrointestinal	5	2
Infection	6	1
Inefficacy/flare	0	7
Other	1	1

## Discussion

In this retrospective, real-world multicenter study evaluating RA patients treated with tofacitinib 5 mg bid for 12 months, this jak-is significantly and rapidly reduced disease activity, with 41.8% of patients achieving LDA, and 13.93% achieving remission according to CDAI at follow up. According to DAS28-CRP, considered most at risk of bias in clinical studies [19], remission and LDA rates were higher: 43.4% and 23% respectively.

Essentially, our results are in line with previous studies confirming the efficacy and safety of tofacitinib in the treatment of RA. Our DAS-28 CRP remission rate was higher than that published in phase I–III clinical trials (7.2%–23.1%) [20–23], while real-world studies agreed with our data [24–26].

The marked improvement in disease activity already at T3 is explained by the rapid mechanism of action of Jaki [24, 27, 28].

The number of failures of bDMARDs influenced changes in DAS28-CRP over time and were predictive of higher values of disease activity.

Patients naive to biologic agents showed a higher remission rate and a shorter time to remission compared to pre-exposed patients. As pointed out by other studies [24, 29, 30], this finding suggests that there may be a benefit in using tofacitinib as a first-line drug, before the initiation of biologic agents.

To our knowledge, this is the first study that reports an increasing of concordance between PROs (PtGA and GH) and PhGA and the subsequent close correlation between DAS-28 and PtGA during a Jaki therapy. Previous studies evaluating agents with different mechanism of action (i.e. anti TNF) highlighted the discordance between patients and physicians in the assessment of global disease activity, and furthermore, DAS-28 on multivariate analysis proved to be independent of PROs [17, 18, 31]. This finding could be consistent with the Tofacitinib and JAKis biochemical mechanism, a dual action that, modulating as inflammation as nociceptive pathways, leads to clinical benefits including a reduction of pain beyond that related to inflammation [32]. Indeed in clinical practice, such as in trial studies with different agents it is not rare to observe a relevant discordance between PtGA and PhGA, because of different illness perception (in particular pain) due to non-inflammatory processes [31].

PROs and PhGA also showed a slight but continuous improvement at T6 and T12 consistent with disease activity, suggesting a long-term modulation of the JAK-STAT pathways, leading to a gradual reduction of the inflammatory burden [3, 33].

No differences in disease activity were observed when patients were categorized by gender and antibody profile.

A total of thirteen patients (10.65%) discontinued tofacitinib, of whom AEs (4.91%) were fewer than the insufficient responses (5.73%).

Although in our database the patient mean age was below the threshold of 65 years (62.3 ± 12.8), indicated by EULAR as a risk factor for JAK-is, 40.9% of patients were ≥ 65 years of age and may represent a group at increased risk. Moreover, at baseline 22.3% were current smokers, 2.45% had a history of MACE, 5.73% had a history of cancer.

Despite the limited sample size, our data regarding safety are consistent with those reported in the literature from RCTs and other real-world cohorts (i.e. AEs incidence rates leading to discontinuation were 5–9% [10, 12, 34] and 4–15% to insufficient clinical response [29, 35]).

Two-recorded MACE (one stroke and one myocardial infarction) did not lead to discontinuation. These two patients were > 75 years, one had already had a myocardial infarction while the other was a past smoker.

Only one case of lymphoma was recorded at T3, consistent with the low malignancy rate reported by an integrated analysis of safety data for tofacitinib [36]. Although it is known from previous works that the incidence of malignancy increases after 18 months [36], so our study probably underestimates this aspect.

Deep vein thrombosis, pulmonary embolism and life-threatening lymphopenia or neutropenia were not observed during the study.

AEs without discontinuation occurred in thirty-two patients. Infections, gastrointestinal intolerance, and blood count alterations, in particular lymphopenia, represented the most common class of overall AEs along with nine herpes zoster reactivations (only one caused definitive discontinuation). This data are consistent with other real-life studies [37].

The loss of follow-up over 12 months, the absence of a control group and the relatively small number of patients enrolled are the main limitations and the lack of a comparison arm are the study's limiting factors; on the other hand, a real life, multicenter patient analysis is a strength point, reflecting the variability of patient population in medical practice.

## Conclusions

Our real-life experience aligns with real-life studies that have already confirmed the tofacitinib efficacy and safety in RA, either in monotherapy or in combination with csDMARDs.

This was the first study that highlight the temporal concordance between PROs, PhGA and disease activity (DAS28) in a European cohort population, maybe due to JAKis mechanism of action.

Furthermore, the efficacy in achieving a rapid remission and/or LDA in naive patients could suggest the possible benefit of using tofactinib in first line.

AEs observed are consistent with literature data and comparable to those observed in other real life studies with TNFi and other biologic DMARDs [38]. No episodes of deaths, DVT or pulmonary embolism were recorded.

As reported by previous studies [10, 36], a consistent number of herpes zoster reactivations was observed, but most cases remained non-serious, allowing tofacitinib continuation after zoster resolution.

Following recent EULAR recommendations, this study is part of a series of real-world studies on JAK-is to assess their safety and efficacy profile. However, further studies are needed to confirm our results.
